# Integrating single-cell and spatial transcriptomics reveals glycolysis heterogeneity and NEK6-mediated progression in colorectal cancer

**DOI:** 10.3389/fimmu.2026.1802329

**Published:** 2026-04-28

**Authors:** Fengming Yang, Yinuo Bian, Yuqi Jin, Yinuo Tan, Tianhao Lao, Jie Yang, Hua Chen

**Affiliations:** 1 Department of Medical Oncology, Key Laboratory of Cancer Prevention and Intervention, Ministry of Education, The Second Affiliated Hospital, Zhejiang University School of Medicine, Hangzhou, China; 2 Zhejiang Provincial Clinical Research Center for Cancer, Hangzhou, China; 3 Cancer Center of Zhejiang University, Hangzhou, China; 4 Center for Medical Research and Innovation in Digestive System Tumors, Ministry of Education, Hangzhou, China; 5 School of Public Health, Nanjing Medical University, Nanjing, China; 6 Department of General Surgery, Affiliated Kunshan Hospital of Jiangsu University, Kunshan, China; 7 Gusu School, Nanjing Medical University, The First People’s Hospital of Kunshan, Kunshan, China

**Keywords:** colorectal cancer, Mendelian randomization, NEK6, single-cell RNA sequencing, spatial transcriptomics

## Abstract

**Background:**

Metabolic reprogramming is a pivotal driver of tumor microenvironment (TME) remodeling and colorectal cancer (CRC) progression. However, the spatial organization of glycolysis heterogeneity and the molecular drivers maintaining the malignant high-glycolytic state remain poorly understood.

**Methods:**

Single-cell RNA sequencing (scRNA-seq), spatial transcriptomics, and Mendelian randomization (MR) analysis were integrated. Glycolytic malignant subtypes were identified using AUCell and Non-negative Matrix Factorization (NMF). Cell-cell communication networks were inferred via CellChat. Pseudotime trajectory analysis and spatial deconvolution were utilized to resolve the developmental dynamics and spatial architecture of subtypes. Finally, key pathogenic regulator genes were screened by integrating large-scale GWAS data with the TCGA cohort, and candidate gene NEK6 was validated through *in vitro* functional assays.

**Results:**

Three distinct malignant subtypes were identified; the Glycolysis-C1 subtype exhibited a high-glycolytic state. Cell-cell communication analysis revealed that Glycolysis-C1 cells function as a dominant signaling hub, secreting MIF ligands that target Macrophages and B cells via the CD74/CD44 receptor complex. Pseudotime analysis traced a lineage trajectory from low-glycolytic C3 to high-glycolytic C1. Spatially, Glycolysis-C1 cells concentrated in tumor domains, functioning as a communication hub at the tumor-stroma interface and remodeling the immune niche via MIF-CD44 signaling. Multi-omics integration and MR analysis identified NEK6 as a candidate gene associated with CRC risk. High NEK6 expression correlated with poor prognosis and an immunosuppressive TME. Functional assays confirmed that NEK6 knockdown significantly suppressed CRC cell proliferation and glycolysis, as well as abrogated M2 macrophage polarization.

**Conclusion:**

This study reveals the spatial and molecular landscape of glycolysis heterogeneity in CRC and identifies NEK6 as a gene functionally associated with the high-glycolytic phenotype. These findings suggest that NEK6 may represent a potential therapeutic vulnerability for future investigation in CRC.

## 1 Introduction

Colorectal cancer (CRC) remains a leading cause of cancer-related mortality worldwide, characterized by substantial intratumoral heterogeneity and a complex tumor microenvironment (TME) (1, 2). Despite advances in therapeutic strategies, the prognosis for advanced CRC varies significantly among patients, largely driven by distinct molecular subtypes and the dynamic interplay between malignant cells and stromal components (3). Traditional bulk RNA sequencing averages gene expression across cell populations, thereby masking the specific contributions of rare but functionally critical cell sub-populations (4). The advent of single-cell RNA sequencing (scRNA-seq) has revolutionized our understanding of the TME, enabling the high-resolution characterization of malignant cell diversity and the identification of specific cell states associated with tumor progression and therapeutic resistance (5, 6).

Metabolic reprogramming, especially the shift to aerobic glycolysis known as the Warburg effect, is a cancer hallmark that promotes rapid proliferation and survival in hypoxic conditions (7). However, metabolic activity within the tumor mass is not uniform. Recent studies utilizing Non-negative Matrix Factorization (NMF) on scRNA-seq data have successfully stratified malignant cells into distinct functional programs, revealing that metabolic heterogeneity drives tumor plasticity (8, 9). Distinct glycolytic malignant states evolve dynamically during tumorigenesis. However, the precise mechanisms by which they spatially remodel the immune microenvironment to establish an immunosuppressive niche remain unclear.

Tumor progression is not merely a static accumulation of mutations but a dynamic spatiotemporal process involving intricate cell-cell communication (10, 11). Pseudotime trajectory analysis offers a computational framework to reconstruct the evolutionary lineage of cancer cells, tracing their transition from early to late metabolic states (12). Furthermore, cellular interactions are inherently spatially constrained. Spatial transcriptomics complements scRNA-seq by preserving the positional context of cells, allowing for the visualization of ligand-receptor interactions, such as those occurring at the tumor-stroma interface (13, 14). Integrating these multi-modal approaches is essential to dissect the specific signaling networks, such as the Macrophage Migration Inhibitory Factor (MIF) pathway, through which metabolically active tumor cells domesticate infiltrating immune cells (15).

While transcriptomic analyzes identify genes correlated with cancer progression, determining causality remains a challenge due to potential confounding factors and reverse causation. Integrating multi-omics data with genetic epidemiology offers a robust solution. Mendelian Randomization (MR), which uses genetic variants as instrumental variables, has emerged as a powerful strategy to screen for candidate genes that are causally associated with disease risk (16, 17). By intersecting differentially expressed genes (DEGs) from scRNA-seq with large-scale Genome-Wide Association Study (GWAS) data, researchers can prioritize therapeutic targets with higher clinical validity (18).

In this study, we integrated scRNA-seq, spatial transcriptomics, and Mendelian randomization analysis to comprehensively map the metabolic and immune landscape of CRC. We identified a high-metabolic malignant subtype (Glycolysis-C1) and reconstructed its developmental trajectory from a low-glycolytic precursor. Cell-cell communication and Spatial analyzes subsequently demonstrated that Glycolysis-C1 cells preferentially localize at tumor-stroma boundaries and reprogram the immune microenvironment via MIF-CD44 signaling.

Multi-omics integration coupled with Mendelian randomization (MR) analysis pinpointed EPB41L2 and NEK6 as causal risk genes contributing to CRC progression. NIMA-related kinase 6 (NEK6), a serine/threonine kinase critical for mitotic progression and spindle formation, has emerged as a key regulator in various malignancies (19–21). Dysregulation of NEK6 is known to disrupt cell cycle checkpoints and promote tumorigenesis, yet its specific role in linking metabolic rewiring to immune evasion in CRC remains to be elucidated. We showed *in vitro* that NEK6 knockdown in CRC cell lines significantly suppressed tumor proliferation, colony formation, and glycolytic activity, while also attenuating M2 macrophage polarization. Collectively, our findings reveal molecular and spatial diversity of glycolysis in CRC and nominate NEK6 as a candidate therapeutic vulnerability for future investigation.

## 2 Materials and methods

### 2.1 Data acquisition and single-cell RNA-seq processing

The raw scRNA-seq data GSE161277 were acquired from the Gene Expression Omnibus (GEO, http://www.ncbi.nlm.nih.gov/geo/) and comprise 50, 061 cells derived from 13 samples across four CRC patients. This cohort partitioning includes 15, 274 cells from tumor tissues, 18, 616 from paratumor and normal tissues, 14, 034 from adenoma tissues, and 2, 137 from peripheral blood. Bulk transcriptomic and clinical data of CRC samples were obtained from The Cancer Genome Atlas (TCGA; https://portal.gdc.cancer.gov/). Additionally, to independently validate prognostic significance, two external CRC cohorts (GSE103479 and GSE17536) along with clinical survival data were downloaded from the GEO database.

The Seurat package was utilized for downstream analysis. Quality control was performed to filter low-quality cells based on the number of detected features and mitochondrial gene percentage. The “Harmony” algorithm was employed to integrate datasets and correct for batch effects among different samples. Principal Component Analysis (PCA) was conducted on highly variable genes, followed by Uniform Manifold Approximation and Projection (UMAP) for dimensionality reduction and visualization.

### 2.2 Identification of malignant epithelial cells

To distinguish malignant epithelial cells from non-malignant cells, chromosomal copy number variations (CNVs) were inferred using the inferCNV package. B cells and Plasma cells served as the normal reference baseline. Initial CNV scores per cell were calculated and visualized via heatmaps, where red and blue indicated chromosomal amplifications and deletions, respectively. The average CNV scores of B and Plasma cells were set as thresholds to exclude non-malignant epithelial cells, retaining malignant epithelial cells for further analysis.

### 2.3 Evaluation of glycolysis activity and metabolic subtypes

To evaluate the metabolic heterogeneity of malignant cells, we utilized the “AUCell” package to score the activity of the glycolysis pathway. The “HALLMARK_GLYCOLYSIS” gene set was retrieved from the Molecular Signatures Database (MSigDB). Subsequently, we applied the Non-negative Matrix Factorization (NMF) algorithm to the expression profiles of these glycolysis-related genes to identify distinct metabolic states. The optimal number of clusters (k=3) was determined by evaluating the cophenetic correlation and dispersion metrics. Unsupervised clustering stratified the malignant cells into three distinct subpopulations, which were sequentially designated as Glycolysis-C1, C2, and C3 according to their mean AUCell scores.

### 2.4 Cell-cell communication analysis

Intercellular communication among Glycolysis cell subtypes and associated immune cells in the CRC tumor microenvironment was characterized using the CellChat package. Interaction assessment relied on matched ligand–receptor expression, employing the Secreted Signaling database with default settings. Visualization utilized ‘netVisual_circle’ to quantify inter-subtype interaction numbers and intensities, and ‘netVisual_bubble’ to depict upregulated ligand–receptor pairs. Subsequently, significant pathways (e.g., MIF signaling) were delineated via chord diagrams to illustrate the detailed network structure.

### 2.5 Single-cell pseudotime analysis

Pseudotime trajectory analysis mapped malignant cell developmental states using the Monocle 2 package (22). The resulting trajectory was constructed via the DDRTree algorithm based on highly variable genes, with the root anchored by the C3 subpopulation’s expression profile. Branched Expression Analysis Modeling (BEAM) then analyzed gene expression dynamics along the trajectory branches.

### 2.6 Spatial transcriptomics processing and analysis

We analyzed the spatial transcriptomics dataset (Accession: GSE283052), which includes 12 colorectal cancer tissue samples, with Seurat to spatially resolve metabolic heterogeneity, the immune microenvironment, and key candidate gene expression. Raw gene expression matrices underwent SCTransform normalization, followed by Principal Component Analysis (PCA) and UMAP dimensionality reduction. Concurrently, we characterized the spatial metabolic landscape by calculating the Glycolysis Activity Score for each spot using UCell, drawing upon the MSigDB Hallmark gene set. Cell-type deconvolution was performed using the SPOTlight (23) algorithm to map the spatial distribution of Glycolysis-C1 subtypes and Macrophages, leveraging marker signatures to estimate cellular proportions per spot.

To investigate intercellular communication *in situ*, spatial ligand-receptor (e.g., MIF-CD44) co-localization analysis was conducted with the SpaGene package (24). An interaction score (LR Score) was calculated to identify potential signaling hotspots, particularly at the tumor-stroma interface. The spatial expression patterns of identified candidate genes were visualized using SpatialFeaturePlot to validate their enrichment in tumor-specific regions.

### 2.7 Identification of DEGs and functional enrichment

To characterize the transcriptomic features of the Glycolysis-C1 subtype, we performed differential expression analysis between Glycolysis-C1 malignant cells and normal epithelial cells using the “FindMarkers” function in Seurat. Genes with an adjusted *P* < 0.05 and |log2FC| > 0.3 were defined as DEGs. Functional enrichment analyzes, including Gene Ontology (GO) and Kyoto Encyclopedia of Genes and Genomes (KEGG), were conducted using the clusterProfiler package to elucidate the biological functions of these DEGs.

### 2.8 Mendelian randomization analysis and multi-omics intersection

To identify candidate genes with potential causal associations with CRC, we performed MR analysis using the TwoSampleMR package. The expression quantitative trait loci (eQTL) exposure data were obtained from the IEU Open GWAS database (25), and the CRC outcome data were retrieved from the GWAS Catalog (Accession: ebi-a-GCST012879). Genetic variants were selected as instrumental variables (IVs) based on genome-wide significance (*P* < 5e-08) and linkage disequilibrium criteria (r² < 0.001, kb = 10000). The Inverse Variance Weighted (IVW) method was employed as the primary analytical approach, supplemented by Weighted Median and MR-Egger methods. Specifically, we evaluated the heterogeneity among the instrumental variables using Cochran’s Q test and assessed directional horizontal pleiotropy via the MR-Egger intercept test.

We intersected three datasets to identify robust candidate genes: DEGs from previous single-cell analysis, DEGs derived from the TCGA-COAD-READ cohort by DESeq2, and genes showing significant causal effects via MR analysis. Filtering involved aligning upregulated DEGs with MR risk factors (OR > 1) and downregulated DEGs with protective factors (OR < 1) to pinpoint key genes.

### 2.9 Immune cell infiltration analysis

To comprehensively characterize the tumor immune microenvironment associated with the candidate genes, we analyzed RNA-seq data from the TCGA-COAD-READ cohort. The relative proportions of 22 distinct immune cell types were quantified using the CIBERSORT algorithm. To ensure the robustness of the infiltration estimation, we also employed the MCP-counter and EPIC algorithms to infer the absolute abundance of immune and stromal cells. Spearman correlation analysis was performed to evaluate the associations between the expression levels of the four candidate genes and the abundance of immune cells. The results were visualized using the linkET package. Furthermore, patients were stratified into high- and low-expression groups based on the median expression of each candidate gene. The differences in immune infiltration profiles across these groups were compared and visualized using heatmaps.

### 2.10 Survival analysis

To evaluate the clinical relevance of the candidate genes, we performed survival analysis using the TCGA-COAD-READ cohort. To further validate the prognostic significance of NEK6, independent validation was conducted using the external GSE103479 and GSE17536 cohorts. Patients were stratified into high- and low-expression groups based on the optimal cutoff value of gene expression. Kaplan-Meier (KM) survival curves were generated, and the log-rank test was used to assess the statistical significance of differences in Overall Survival (OS). A P-value < 0.05 was considered statistically significant.

### 2.11 Validation of protein expression

To validate the protein expression levels of the candidate genes in colorectal cancer tissues, we retrieved immunohistochemistry (IHC) staining images from the Human Protein Atlas (HPA) database (https://www.proteinatlas.org/). Representative images comparing tumor and normal tissues were selected.

### 2.12 Cells and culture conditions

Human colorectal cancer cell lines (HCT116, SW480), and human monocytic leukemia cell line (THP-1) were sourced from the American Type Culture Collection (ATCC). The cells were grown in DMEM/RPMI-1640 medium with 10% fetal bovine serum and 1% penicillin-streptomycin, maintained at 37˚C in a 5% CO_2_.

### 2.13 Cell transfection

The siRNAs specific to NEK6 (si-NEK6-1, si-NEK6-2, and si-NEK6-3) were predesigned and screened for NEK6 inhibition in CRC cells, with a negative control siRNA (si-NC) designed from a nonhuman homologous sequence. Lipofectamine 3000 (Thermo Fisher Scientific) was utilized for siRNA transfection in accordance with the manufacturer’s protocols. The siRNA sequences were as follows: si-NEK6-1: 5′- AGUUCAGGGCCUTTAUCTTTG-3′; si-NEK6-2: 5′-GCGGTUACAATTTCCACGAGT-3′; si-NEK6-3: 5′-GCAATTTUGCCAACGTTGATA-3′. Based on the results of RT-qPCR and western blot analysis, si-NEK6–2 showed the highest knockdown efficiency and was therefore selected for all subsequent functional assays.

### 2.14 Real-time quantitative polymerase chain reaction

The mRNA expression of NEK6 was assessed using RT-qPCR. Total RNA was isolated from CRC cell lines with TRIzol reagents. The qPCR procedure started with a 10-minute phase at 95 °C, then 40 cycles of amplification at 95 °C for 15 seconds, 60 °C for 60 seconds, and 95 °C for 1 second. The reverse transcription process was carried out at 25 °C for 10 minutes, followed by 42 °C for 15 minutes, and then 85 °C for 5 minutes. The sequences of the oligonucleotides in this study were as follows: NEK6, forward, 5’- TCTTGAAGCAACTGAACCA-3’ and reverse, 5’- CCAACTCCAGCACAATGT-3’; GAPDH, forward, 5’-GAGAAGGCTGGGGCTCATTT-3’ and reverse, 5’-ATGACGAACATGGGGGCATC-3’.

### 2.15 Western blot assay

Cells were lysed using a standard solution, and the resulting total protein concentrations were quantified via the BCA assay. Proteins were then resolved using SDS-PAGE electrophoresis and subsequently electrotransferred onto a PVDF membrane. Following a 15-minute rinse in Tris-buffered saline (TBS), the membrane was blocked and incubated overnight at 4 °C with the primary antibodies: Anti-NEK6, Anti-LDHA, Anti-GLUT1, and β-actin. Secondary antibody incubation proceeded for 2 hours at room temperature, after which bands were identified using an enhanced chemiluminescence (ECL) reagent.

### 2.16 Cell proliferation assay

Cell proliferation was quantified using the Cell Counting Kit-8 (CCK-8). For initial seeding, approximately 5×10³ cells were plated per well in 96-well plates and cultured in complete medium. At 0, 24, 48, and 72 hours post-transfection, 10 μL of CCK-8 solution was introduced to each well, followed by incubation at 37 °C for 2–4 hours. Absorbance (OD_450_) was subsequently read using a microplate reader. All measurements were performed in triplicate.

### 2.17 Colony formation assay

HCT116 and SW480 cells were seeded in triplicate at a density of 500 cells per well in 6-well plates in complete growth medium. Following 14 days of incubation, cells were fixed with 100% methanol (15 minutes) and subsequently stained using 0.5% crystal violet solution (30 minutes). Once air-dried, colonies exceeding 50 cells were manually enumerated.

### 2.18 Glycolysis stress test and detection of lactate production and ATP levels

The Extracellular Acidification Rate (ECAR) test was used to estimate metabolic alterations in transfected HCT116 and SW480 cells by using Seahorse Bioscience XF-24 Extracellular Flux Analyzer. The basic glycolysis levels of transfected cells were analyzed using Wave 2.3 software. Lactate production and ATP concentrations in transfected HCT116 and SW480 cells were assessed using the Lactic Acid Content Assay Kit and the ATP Assay Kit, respectively. All assays followed the manufacturer’s instructions.

### 2.19 THP-1 macrophage co-culture and flow cytometry

To induce differentiation into M0 macrophages, THP-1 cells were treated with 100 ng/mL phorbol 12-myristate 13-acetate (PMA) for 48 hours. Subsequently, the PMA-containing medium was replaced with fresh medium, and the THP-1-derived macrophages were allowed to rest for 24 hours. For the *in vitro* co-culture system, these differentiated macrophages were cultured in the lower chambers of Transwell inserts, while CRC cells transfected with si-NC or si-NEK6 were seeded in the upper chambers. Macrophages cultured alone without CRC cells served as a blank control. After a 48-hour co-culture, lower-chamber macrophages were collected, washed twice with chilled PBS, and resuspended in staining buffer. The cells were then incubated with fluorescently conjugated anti-CD68 and anti-CD206 antibodies at 4˚C in the dark for 30 minutes. Phenotypic polarization was subsequently quantified via flow cytometry and analyzed using FlowJo software. The blank group represented a biological control consisting of macrophages cultured alone under the same staining conditions, while the full gating strategy used for analysis is provided in the **Supplementary Figure 3**.

### 2.20 Statistical analysis

Statistical analyzes and data processing were conducted using R (v4.4.1) and GraphPad Prism 10.4. Continuous variables were summarized based on distribution: normally distributed data are reported as mean ± standard deviation (SD), whereas skewed distributions are presented as median (interquartile range, IQR). Group comparisons employed the independent samples t-test and the Wilcoxon rank-sum test. Statistical significance was defined at *P* < 0.05, with the following conventional notation applied: *P < 0.05; **P < 0.01; ***P < 0.001; and ****P < 0.0001.

## 3 Results

### 3.1 Single-cell transcriptomic landscape of colorectal cancer

**Figure 1:**
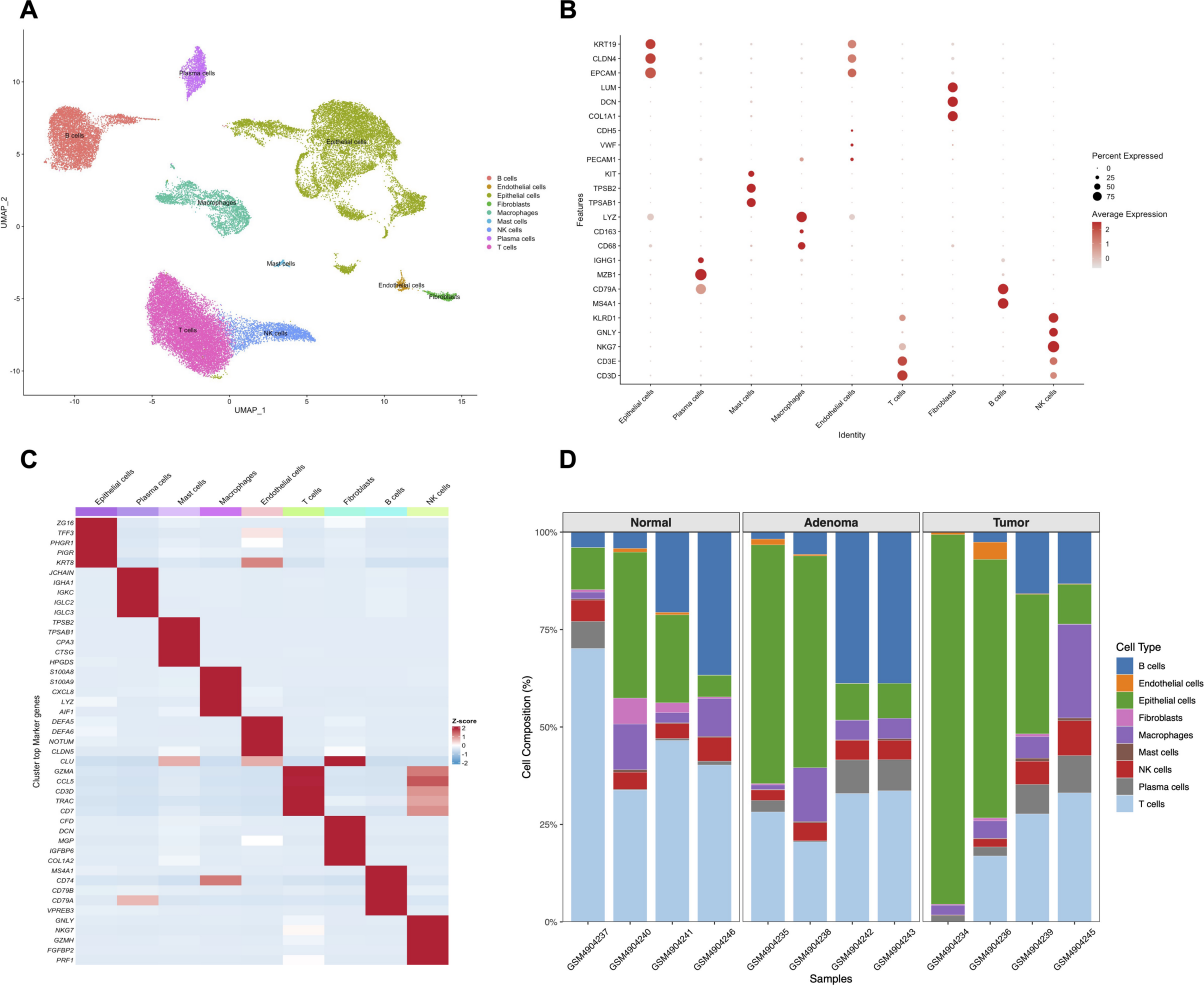
The single-cell transcriptomic landscape of CRC. **(A)** UMAP plot visualizing the 9 major cell types identified in the CRC tumor microenvironment. Each point represents a single cell, colored by its annotated cell type. **(B)** Dot plot showing the expression levels of canonical marker genes across the identified cell clusters. The dot size represents the percentage of cells expressing the gene, and the color intensity represents the average expression level. **(C)** Heatmap displaying the top differentially expressed marker genes for each cell type. **(D)** Stacked bar plot illustrating the proportional distribution of the cell types across individual samples.

To dissect the cellular heterogeneity within the tumor microenvironment of CRC, we analyzed scRNA-seq data from the GSE161277 cohort. After strict quality control and batch correction using Harmony, unsupervised clustering was performed. Based on the expression of canonical lineage markers, we successfully identified and annotated 9 major cell types across the 13 samples, including B cells, Plasma cells, T cells, NK cells, Macrophages, Mast cells, Fibroblasts, Epithelial cells, and Endothelial cells (**Figure 1A**). The accuracy of the cell type annotation was validated by the expression patterns of specific marker genes, as shown in the dot plot (**Figure 1B**) and heatmap (**Figure 1C**). Furthermore, we analyzed the cellular composition across different samples. The stacked bar plot illustrated the relative proportions of these cell types across patient samples (**Figure 1D**), revealing inter-tumoral heterogeneity in the TME composition.

### 3.2 Identification of malignant cells and glycolysis subtypes

**Figure 2:**
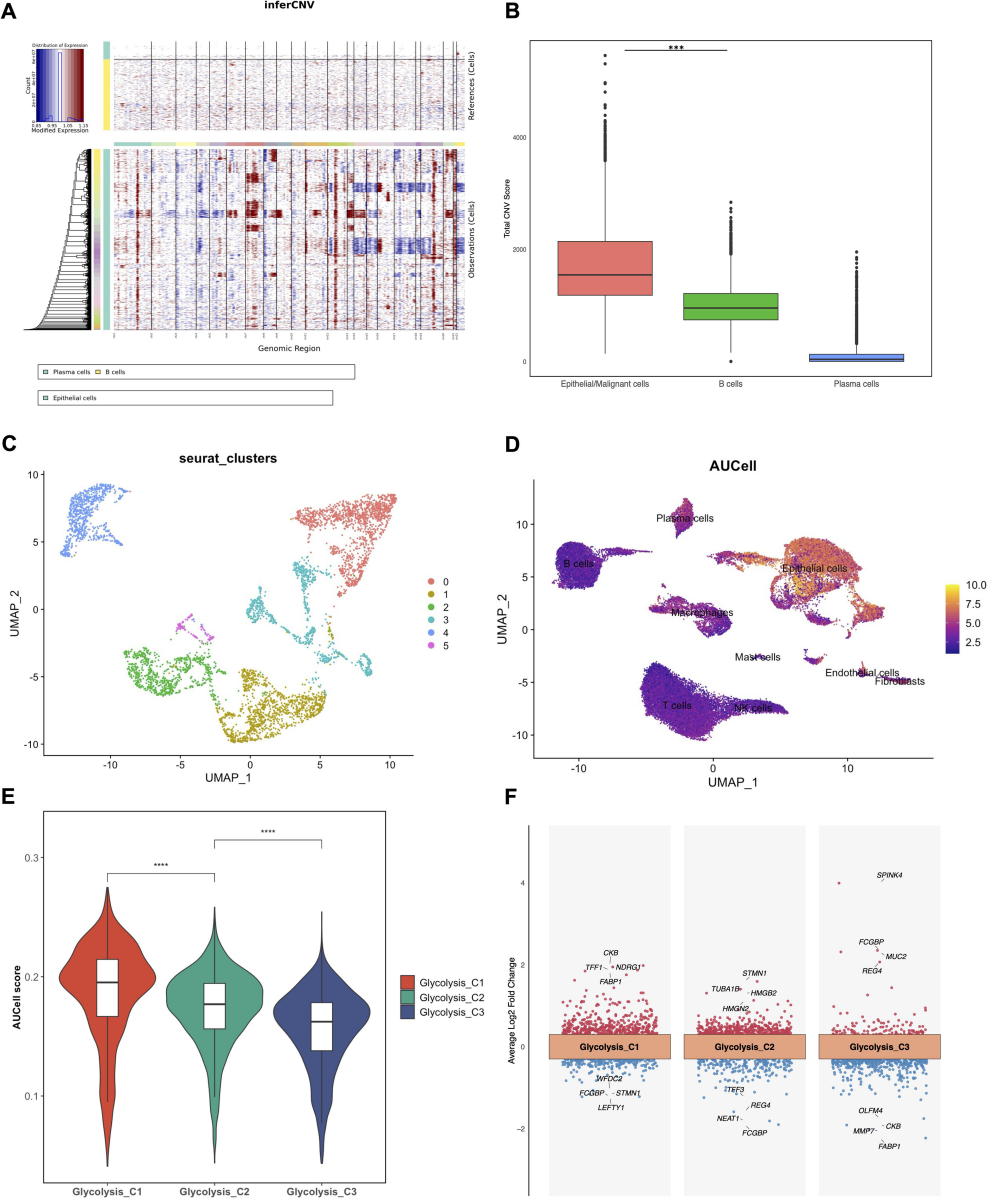
Identification of malignant cells and glycolysis subtypes. **(A)** Heatmap of large-scale chromosomal copy number variations inferred by InferCNV. **(B)** Boxplot comparing the total CNV scores between Epithelial/Malignant cells and reference immune cells. ***P < 0.001. **(C)** UMAP plot showing the re-clustering of epithelial cells into 6 clusters. **(D)** UMAP plot visualized by the AUCell score showing Glycolysis heterogeneity. **(E)** Violin plot illustrating the significant differences in glycolysis scores among the three NMF-identified malignant Glycolysis subtypes. ****P < 0.0001. **(F)** Scatter plot displaying the top differentially expressed marker genes for each glycolysis-associated malignant subtype.

To distinguish malignant epithelial cells from normal cells, InferCNV analysis was performed using B and Plasma cells as a reference and demonstrated widespread chromosomal copy number variations (CNVs) in the epithelial population of the tumor group (**Figure 2A**). Consistently, the total CNV scores of the tumor group epithelial cells were significantly higher than those of the reference immune cells (**Figure 2B**). To further refine the classification, we re-clustered the tumor group epithelial cells into six sub-clusters (Clusters 0–5) (**Figure 2C**). By integrating the CNV scores, Cluster 4 was identified as normal epithelial cells due to its genomic stability, while the remaining clusters (0, 1, 2, 3, and 5) were identified as malignant cells.

Given the critical role of metabolic reprogramming in cancer, we assessed the glycolysis activity of individual cells using the AUCell algorithm based on the MSigDB Hallmark gene set. The UMAP revealed substantial heterogeneity in glycolysis levels across the TME (**Figure 2D**). Focusing on the malignant cells, we applied NMF clustering to stratify them into three distinct metabolic subtypes: Glycolysis-C1, Glycolysis-C2, and Glycolysis-C3. As shown in **Figure 2E**, the Glycolysis-C1 subtype exhibited the highest glycolysis score, suggesting a high-metabolic state, followed by C2 and C3. Differential expression analysis highlighted specific marker genes for each subtype (**Figure 2F**).

### 3.3 Cell-cell communication between glycolysis subtypes and immune cells

**Figure 3:**
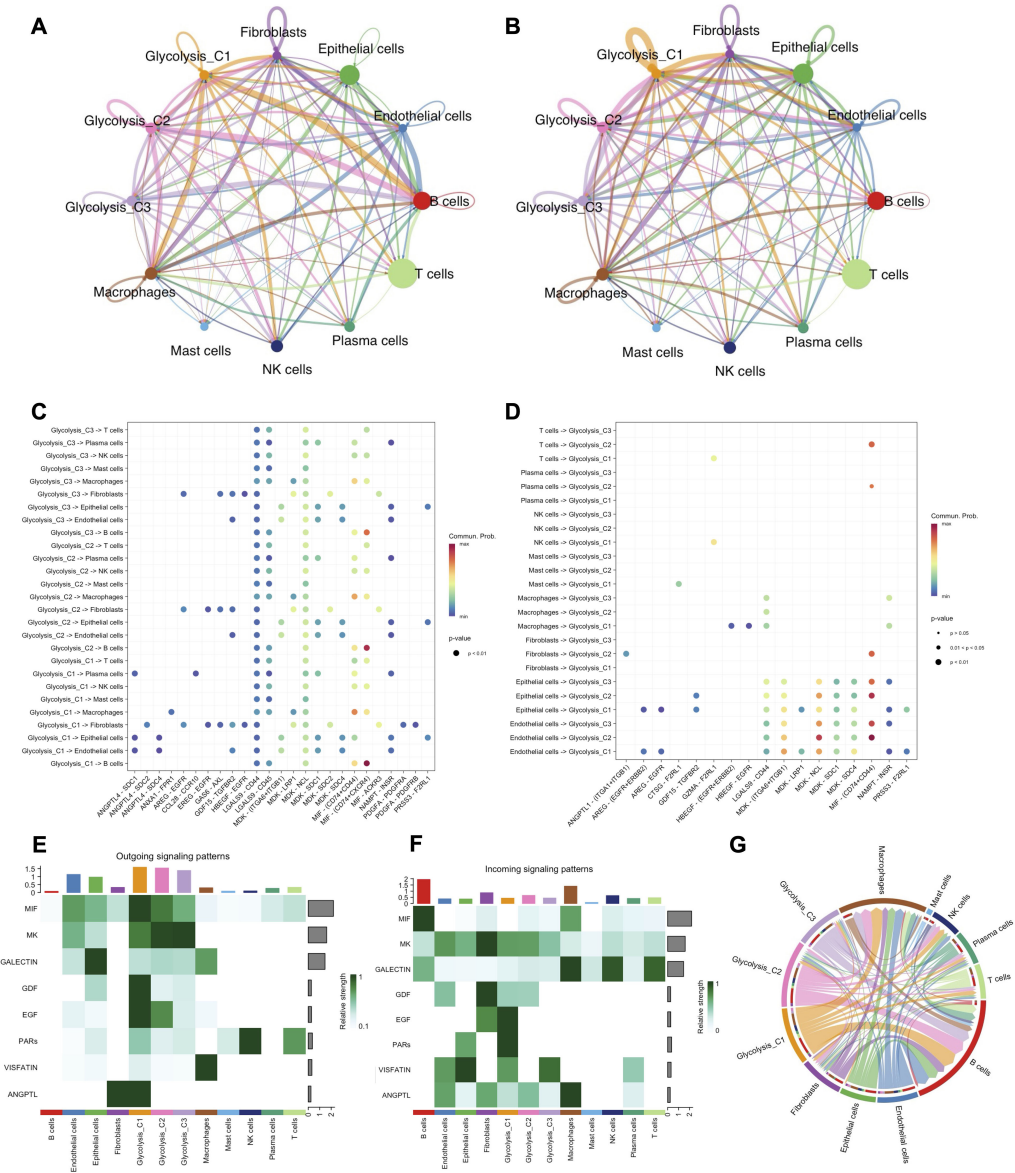
Cell-cell communication between glycolysis subtypes and immune cells. **(A–B)** Circle plots visualizing the total number of interactions **(A)** and interaction weights/strength **(B)** between the three glycolysis-associated malignant subtypes and other TME cell types. **(C)** Bubble map showing significant outgoing ligand-receptor pairs originating from the Glycolysis subtypes targeting other cell types. **(D)** Bubble map showing significant incoming ligand-receptor pairs targeting the Glycolysis subtypes from other cell types. **(E)** Heatmap of outgoing signaling patterns, identifying Glycolysis-C1 as a major sender for MIF pathways. **(F)** Heatmap of incoming signaling patterns, showing how different pathways are received by cell types. **(G)** Chord diagram visualizing the MIF signaling pathway network.

To elucidate the molecular crosstalk between malignant metabolic subtypes and the immune microenvironment, we constructed a cell-cell communication network using CellChat. The aggregate network analysis revealed a complex interaction landscape, with distinct variations in the number and strength (**Figures 3A, B**) of interactions among the three glycolysis-associated subtypes and stromal/immune cells.

We further dissected the specific signaling directions. The bubble plot of outgoing signaling patterns (**Figure 3C**) demonstrated that Glycolysis-C1 cells were primary signal sources, engaging robustly with Macrophages and B cells via multiple ligand-receptor pairs, including MIF-(CD74+CD44/CXCR4). In contrast, incoming signaling analysis (**Figure 3D**) indicated these Glycolysis subtypes also function as receivers, accepting signals such as MDK-NCL from endothelial and epithelial cells. Pattern recognition analysis confirmed that Glycolysis-C1 was the dominant source of MIF and ANGPTL pathway signals (**Figure 3E**). The MIF pathway signal was widely received by multiple cell types, including B cells and Macrophages (**Figure 3F**). Given the enrichment of MIF in both outgoing and incoming patterns, we specifically visualized the MIF signaling network (**Figure 3G**). The chord diagram highlighted that Glycolysis-C1 cells serve as the primary hub, secreting MIF ligands that predominantly interact with Macrophages and B cells, potentially fostering an immunosuppressive or pro-tumorigenic niche.

### 3.4 Pseudotime trajectory analysis characterizes the metabolic evolution of malignant cells

**Figure 4:**
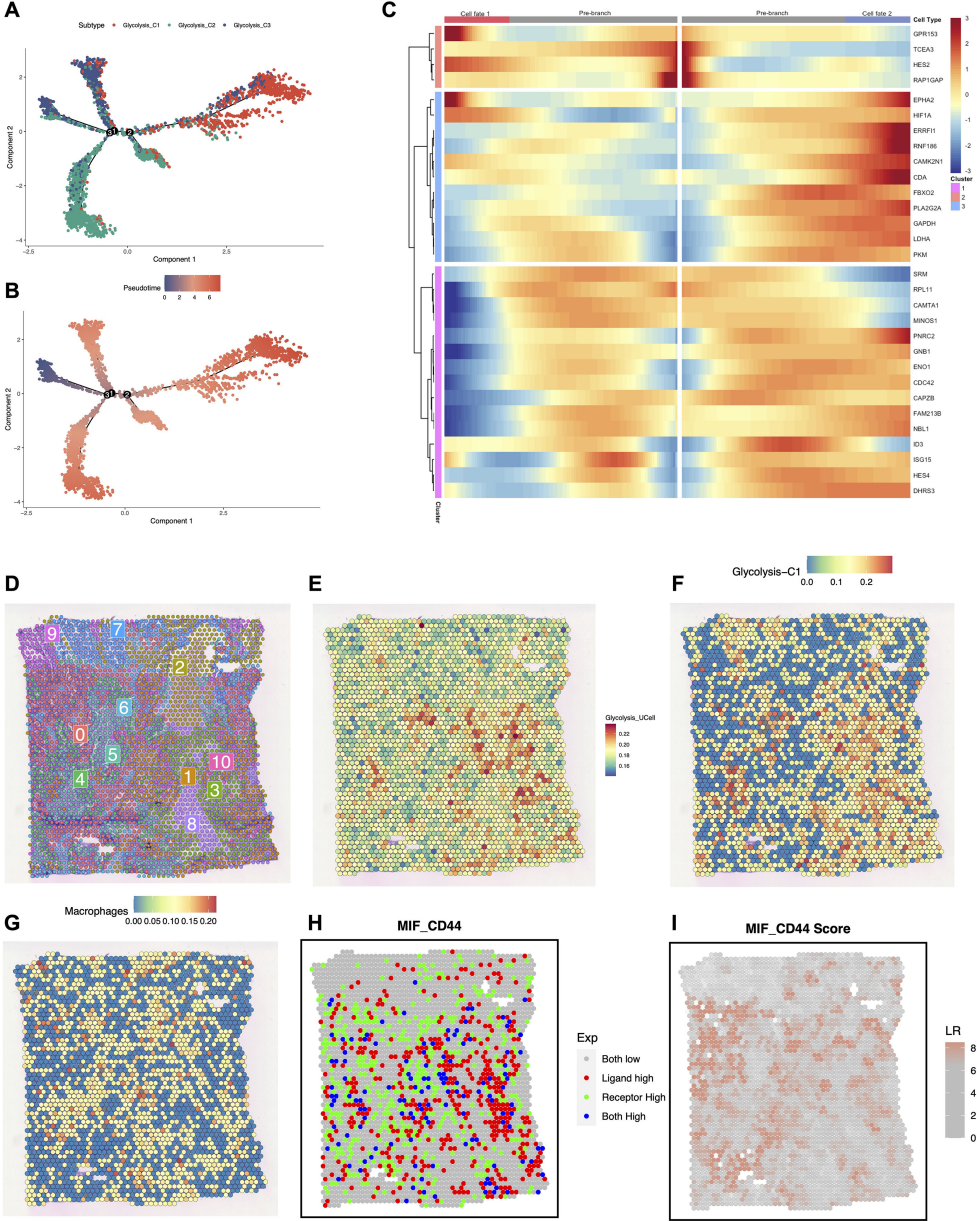
Pseudotime trajectory and spatial transcriptomics analysis of metabolic heterogeneity and ligand-receptor interactions. **(A)** Semi-supervised trajectory inferred by Monocle 2. **(B)** Trajectory colored by pseudotime, indicating the progression from early to late states. **(C)** The branched heatmap of dynamic genes driving differentiation toward cell fates 1 and 2. **(D)** Cluster plot of 0–10 subgroups clustered by Seurat. **(E)** Spatial distribution of the Glycolysis Activity Score. **(F, G)** Spatial plot showing the distribution of Glycolysis-C1 cells and Macrophages predicted by SPOTlight. **(H, I)** Spatial mapping of the MIF ligand, CD44 receptor, and their binding score interaction analysis.

To explore the developmental relationship among metabolic subtypes, we performed single-cell pseudotime trajectory analysis using Monocle 2. The trajectory reconstruction revealed that malignant cells were distributed along a main axis with a trifurcated structure (**Figure 4A**). Mapping the metabolic subtypes onto this trajectory showed that the Glycolysis-C3 (Low) subpopulation was concentrated at the root state, while the Glycolysis-C2 (Intermediate) subpopulation occupied the transitional region. The Glycolysis-C1 (High) subpopulation was predominantly located at the terminal branch (**Figure 4A**). Consistently, pseudotime coloring indicated a continuous progression from early (dark blue) to late (red) developmental states (**Figure 4B**). Branched Expression Analysis Modeling (BEAM) further identified gene expression changes associated with this lineage bifurcation (**Figure 4C**). Specifically, core glycolytic genes (e.g., LDHA, GAPDH) and hypoxia-response genes were highly expressed in the lineage leading to the C1 phenotype, suggesting metabolic adaptation to hypoxia during tumor progression.

### 3.5 Spatial distribution of glycolysis subtypes and ligand-receptor interactions

We subsequently investigated the spatial organization of these metabolic subtypes and their potential interactions with the immune microenvironment. Unsupervised spatial clustering identified distinct tissue domains consistent with histological structures (**Figure 4D**). The spatial distribution of the Glycolysis Activity Score (UCell) showed considerable heterogeneity, with high-scoring regions forming localized clusters (**Figure 4E**). Deconvolution analysis using SPOTlight revealed that these high-metabolic regions were populated by the Glycolysis-C1 subtype (**Figure 4F**). In comparison, Macrophages were distributed in the stromal regions adjacent to the C1-enriched tumor cores (**Figure 4G**, **Supplementary Figure 1**), exhibiting a pattern of spatial adjacency. To characterize potential cell-cell communication at this interface, we analyzed the MIF-CD44 ligand-receptor pair using SpaGene. The categorical map showed spatial co-localization of MIF and CD44 expression (**Figure 4H**, Blue dots), and the interaction score map displayed elevated signaling intensity at the tumor-stroma boundary (**Figure 4I**).

### 3.6 Identification of causal candidate genes via multi-omics integration

**Figure 5:**
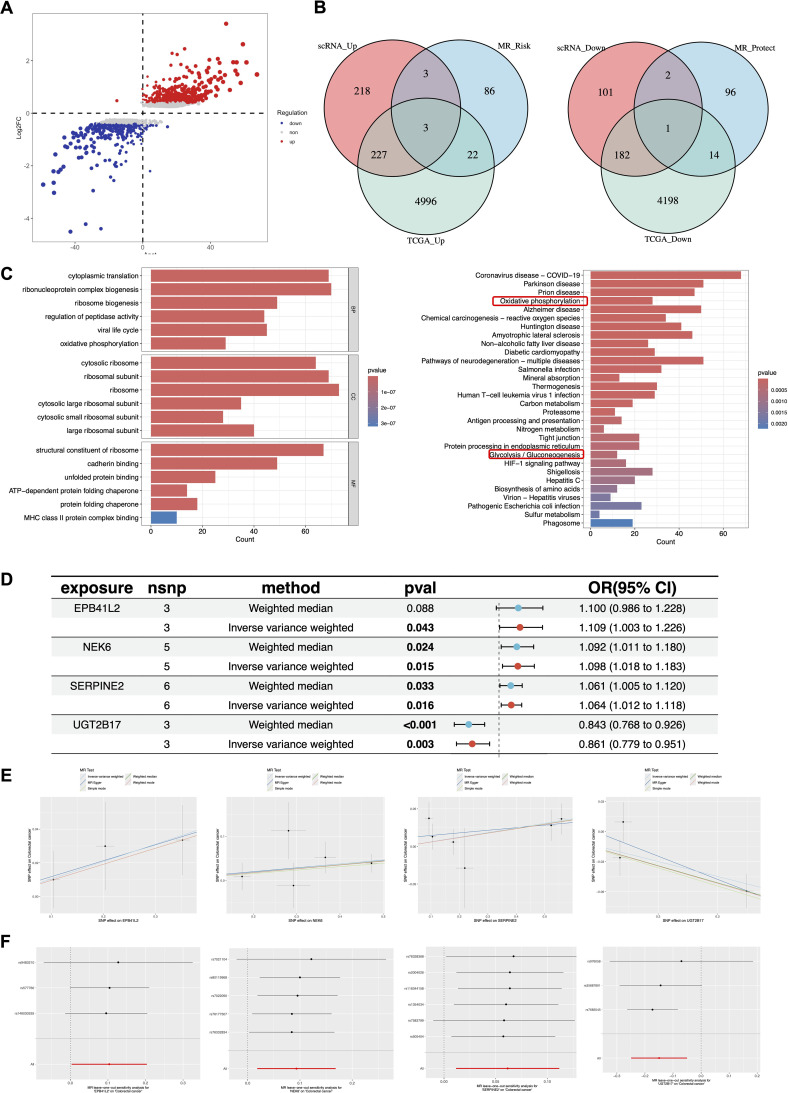
Identification of causal candidate genes via multi-omics integration. **(A)** Volcano plot of DEGs between Glycolysis-C1 malignant cells and normal epithelial cells. **(B)** Venn diagrams showing the identification of 4 candidate genes by intersecting scRNA-seq, TCGA, and MR-identified causal genes. **(C)** Bar plots showing the Gene Ontology (GO) and Kyoto Encyclopedia of Genes and Genomes (KEGG) enrichment analysis of the DEGs. **(D)** Forest plot summarizing the risk estimates for the 4 candidate genes. **(E)** Scatter plots of SNP-level MR results, illustrating the effect of gene expression on colorectal cancer risk. **(F)** Leave-one-out sensitivity analysis plots, demonstrating the robustness of the causal associations for the 4 candidate genes.

To uncover the molecular drivers specific to the Glycolysis-C1 subtype, we first compared its gene expression profile with that of normal epithelial cells. A total of 737 DEGs were identified, including 451 upregulated and 286 downregulated genes (**Figure 5A**). Functional enrichment analysis revealed that these DEGs were significantly enriched in metabolic pathways, particularly Oxidative phosphorylation and Glycolysis/Gluconeogenesis (**Figure 5C**), reaffirming the high-metabolic nature of the C1 subtype.

To further narrow down genes with causal pathogenic potential, we integrated our scRNA-seq data with bulk RNA-seq data from the TCGA-COAD/READ cohort and large-scale GWAS data for Mendelian Randomization (MR) analysis. By intersecting the upregulated DEGs from both scRNA-seq and TCGA with MR-identified risk factors (OR > 1), we identified three potential oncogenes: EPB41L2, NEK6, and SERPINE2. Conversely, intersecting the downregulated DEGs with MR-identified protective factors (OR < 1) revealed one potential tumor suppressor: UGT2B17 (**Figure 5B**).

The MR analysis results for these four candidate genes are detailed in **Figure 5D**. EPB41L2, NEK6, and SERPINE2 showed a positive causal association with CRC risk (*P* < 0.05, OR > 1), while UGT2B17 exhibited a protective effect (*P* < 0.05, OR < 1). The scatter plots visualized the consistent effects of individual SNPs on gene expression and CRC risk (**Figure 5E**). Furthermore, leave-one-out sensitivity analysis confirmed the robustness of these causal associations, indicating that the results were not driven by any single genetic variant (**Figure 5F**). The detailed statistics for the heterogeneity and pleiotropy tests are provided in **Supplementary Table 1**.

### 3.7 Immune cell infiltration analysis of candidate genes

**Figure 6:**
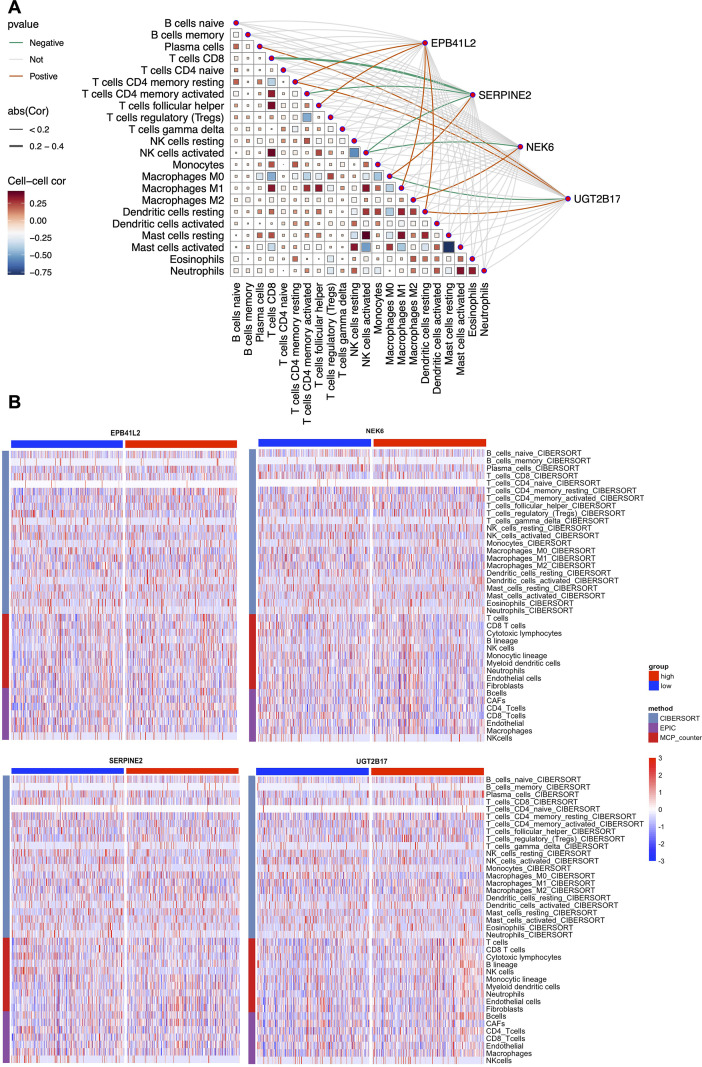
Immune cell infiltration analysis of candidate genes. **(A)** Correlation heatmap visualizing the relationship between the expression of 4 candidate genes and the infiltration levels of 22 immune cell types. **(B)** Heatmaps depicting the differential immune infiltration profiles between high- and low-expression groups of the four candidate genes, as quantified by three algorithms.

To explore the potential role of the identified candidate genes in remodeling the tumor microenvironment, we correlated their expression with immune cell infiltration in the TCGA cohort. As shown in **Figure 6A**, the correlation heatmap revealed distinct immunomodulatory profiles for the risk and protective genes. The risk gene NEK6 was positively associated with M2 Macrophages and negatively associated with NK cells. Similarly, SERPINE2 expression was inversely correlated with both CD8^+^T cells and NK cells. In contrast, the protective gene UGT2B17 displayed a distinct correlation pattern, potentially associating with a more favorable immune phenotype.

Stratification of patients based on gene expression, followed by immune deconvolution using three algorithms (CIBERSORT, EPIC, MCP-counter), showed that high-risk gene expression correlates with an immunosuppressive microenvironment (**Figure 6B**). Conversely, UGT2B17 groups displayed an opposite immune profile. These data imply that these genes may drive CRC progression by regulating the recruitment and polarization of specific TME components.

### 3.8 Prognostic value and external validation of candidate genes

**Figure 7:**
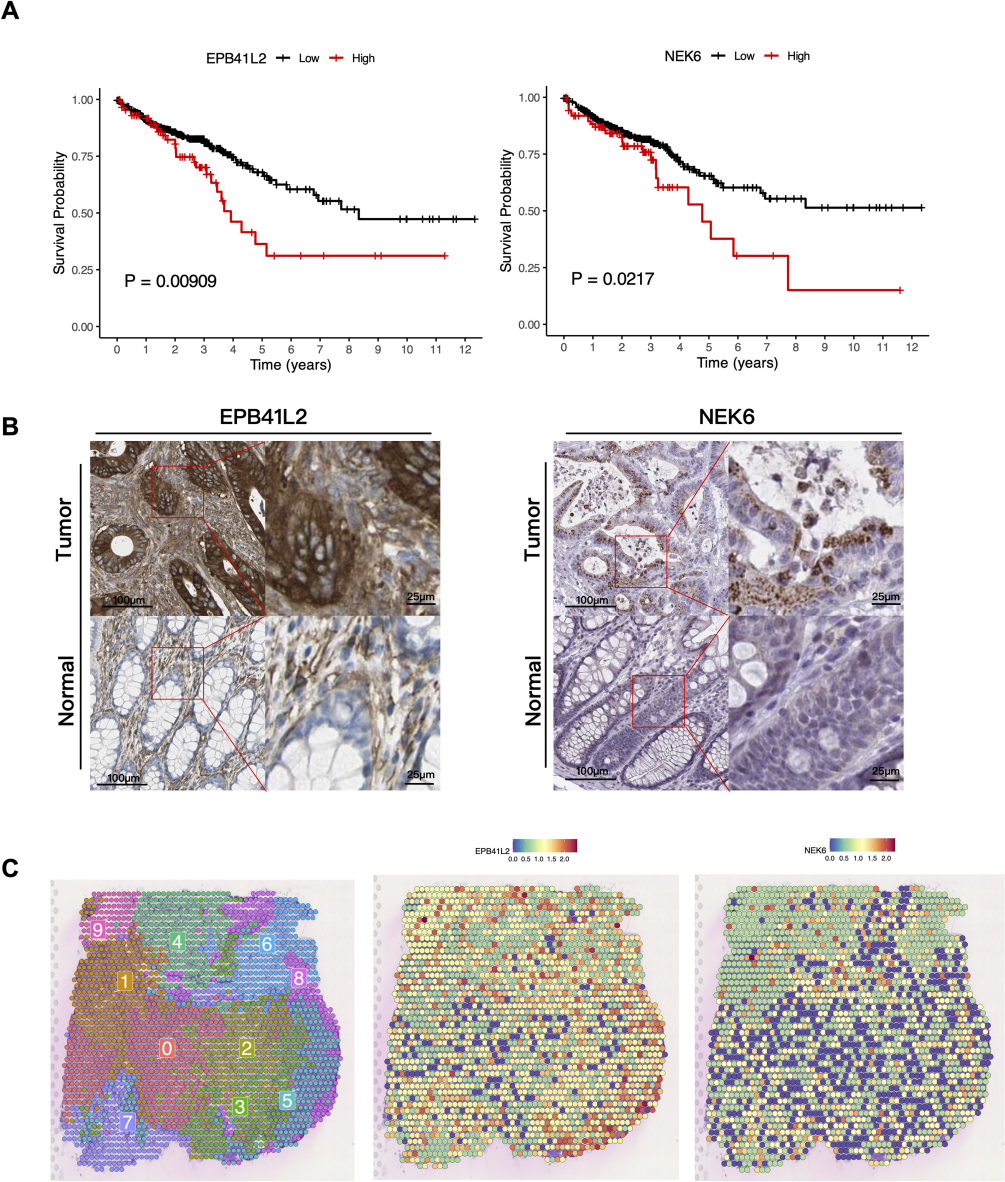
Prognostic value and external validation of candidate genes. **(A)** Kaplan-Meier survival curves for OS based on the expression of EPB41L2 and NEK6 in the TCGA colorectal cancer cohort. **(B)** Representative IHC images from the HPA database. **(C)** Spatial visualization of EPB41L2 and NEK6 in the GSE283052 dataset.

To determine the prognostic significance of the four candidate genes, we conducted Kaplan-Meier survival analysis in the TCGA cohort. As shown in **Figure 7A**, high expression of EPB41L2 (*P* = 0.00909) and NEK6 (*P* = 0.0217) was significantly associated with poorer OS, indicating their potential roles as risk factors and adverse prognostic biomarkers in CRC. We further validated the protein expression of the significant risk genes using IHC data from the HPA database. Consistent with the transcriptomic findings, EPB41L2 and NEK6 proteins were strongly stained in tumor tissues compared to normal colon tissues (**Figure 7B**). Finally, to corroborate these findings in a spatial context, we mapped the expression of EPB41L2 and NEK6 onto the spatial transcriptomics slides. The spatial feature plots revealed that both genes were highly expressed in tumor-enriched regions (**Figure 7C**). The involvement of EPB41L2 in colorectal cancer has been previously reported (26, 27), whereas the role of NEK6 remains unexplored. To validate the clinical prognosis of NEK6, survival analysis was extended to independent cohorts (GSE103479, GSE17536), confirming that elevated NEK6 expression correlates with poor survival. Spatial mapping across 12 samples localized NEK6 enrichment within Glycolysis-C1 niches (**Supplementary Figure 2**), suggesting its association with a high-glycolytic tumor microenvironment.

### 3.9 Downregulation of NEK6 inhibits CRC cell proliferation, glycolysis, and M2 macrophage polarization

**Figure 8:**
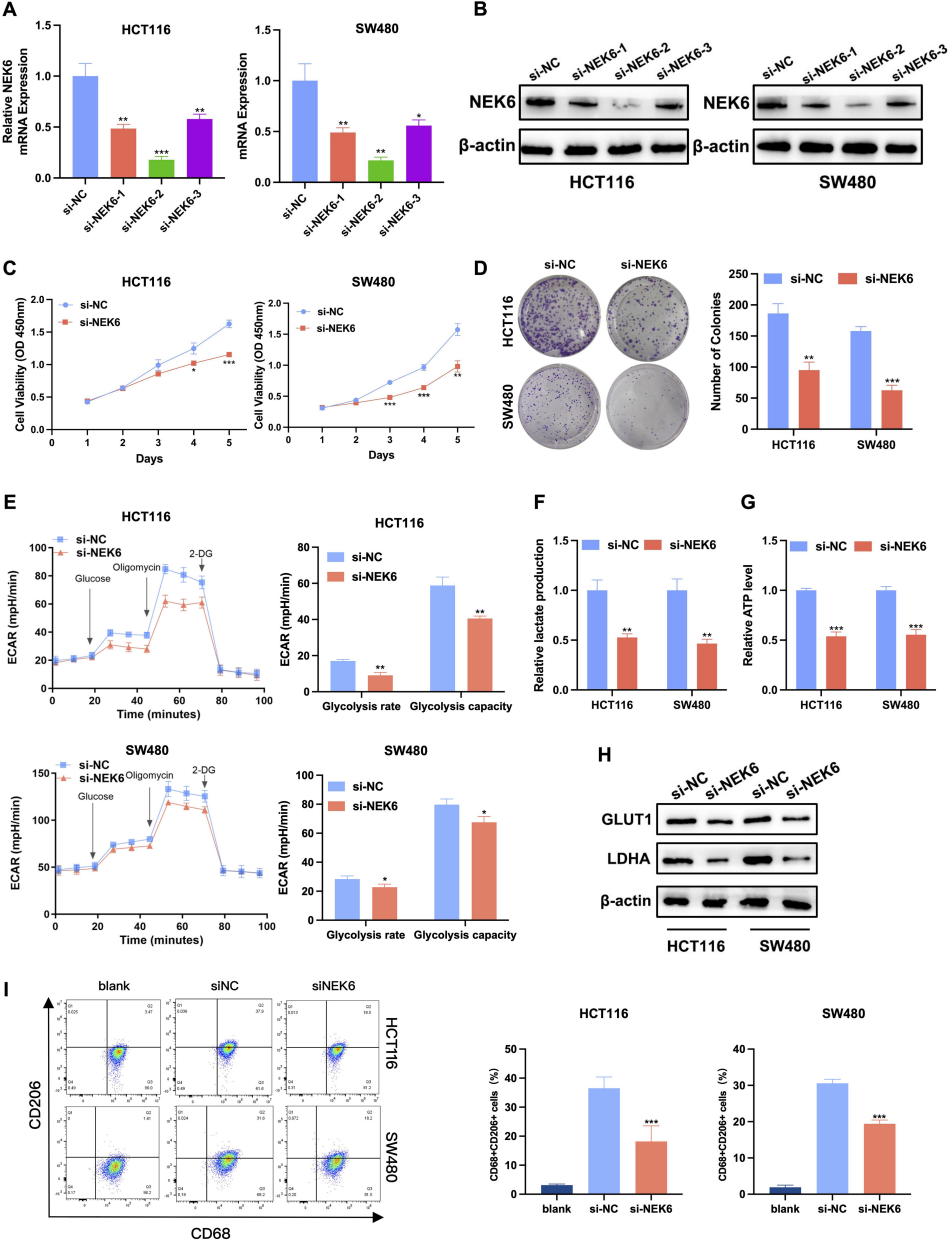
Downregulation of NEK6 inhibits CRC cell proliferation, glycolysis, and M2 macrophage polarization. **(A, B)** Transfection efficiency of NEK6 siRNA was measured by RT-qPCR and western blot. **(C)** CCK8 assay showed the effect of NEK6 on the proliferation rate of HCT116 and SW480 cells. **(D)** Colony formation assay showed the effect of NEK6 on the colony-forming ability of HCT116 and SW480 cells. **(E)** The metabolic profile of NEK6-knockdown CRC cells was assessed by monitoring the ECAR and quantifying both lactate production **(F)** and ATP levels **(G)**. **(H)** Protein levels of LDHA and GLUT1 in NEK6 knockdown HCT116 and SW480 cells. **(I)** Representative flow cytometry plots and quantitative analysis of CD206+ (M2) macrophages. THP-1-derived macrophages were cultured alone (blank) or co-cultured with CRC cells transfected with si-NC or si-NEK6.

To investigate the potential involvement in colorectal cancer cell proliferation, we transfected HCT116 and SW480 cell lines with siRNA targeting NEK6. The knockdown efficiency was confirmed by RT-qPCR and western blot (**Figures 8A, B**). Among them, si-NEK6–2 exhibited the greatest suppression of NEK6 expression at both the mRNA and protein levels in CRC cells, and was used for all subsequent *in vitro* assays. Compared with the negative control, knockdown of NEK6 significantly inhibited cell proliferation, as demonstrated by CCK-8 and colony formation assays (**Figures 8C, D**). NEK6 knockdown effects on glycolytic metabolism in CRC cells were assessed by measuring the ECAR. The downregulation significantly diminished ECAR levels, concurrent with a marked reduction in lactate and ATP production, across both HCT116 and SW480 cell lines (**Figures 8E–G**). Western blot analysis revealed that NEK6 knockdown prominently decreased the protein levels of LDHA and GLUT1 in both cell lines (**Figure 8H**). To validate the impact of NEK6 on the immune microenvironment, we established a co-culture system using THP-1-derived macrophages and CRC cells. Flow cytometry analysis revealed that co-culture with CRC cells promoted the expression of the M2 macrophage marker CD206, whereas NEK6 knockdown abrogated this effect (**Figure 8I**, **Supplementary Figure 3**). These functional data are consistent with our single-cell and spatial analyzes, supporting an association between NEK6 expression and glycolytic as well as immunomodulatory phenotypes in CRC.

## 4 Discussion

In this study, we systematically dissected the metabolic heterogeneity of colorectal cancer by integrating single-cell RNA sequencing, spatial transcriptomics, and Mendelian randomization. We identified a high-metabolic malignant subpopulation, Glycolysis-C1, that is associated with tumor progression and immune remodeling. Unlike previous studies that often viewed tumor metabolism as a uniform characteristic (8), our high-resolution analysis revealed a progressive lineage trajectory where malignant cells evolve from a low-metabolic state to a high-glycolytic phenotype. Importantly, our integrative analyzes and *in vitro* loss-of-function experiments support a functional association between NEK6 and this high-glycolytic phenotype. Furthermore, we mapped the spatial architecture of these cells and found evidence consistent with a paracrine interaction in which Glycolysis-C1 cells may remodel the immune microenvironment via the MIF-CD44 axis at the tumor-stroma interface.

Metabolic Heterogeneity and Tumor Evolution Metabolic reprogramming, particularly the “Warburg effect, ” is a hallmark of cancer (7). However, the spatial organization and intrinsic intra-tumoral heterogeneity of this process remain understudied. Our identification of three distinct metabolic subtypes demonstrates that malignant cells contribute unequally to the tumor’s overall metabolic burden. The pseudotime trajectory suggests the C1 subtype represents a terminal, aggressive state arising from selective pressure. The upregulation of hypoxia-response genes within the C1 lineage directly supports the concept that hypoxic niches drive metabolic adaptation and subsequent aggressiveness (28). Moreover, the extensive copy number variations (CNVs) observed in the C1 subtype suggest a functional link between metabolic stress and chromosomal instability (CIN). This is consistent with recent evidence suggesting that glycolytic intermediates can influence chromatin remodeling and genome stability, creating a feed-forward loop that accelerates tumor evolution (29).

While highly glycolytic tumors are known to generate acidic microenvironments that suppress T-cell function and promote M2 macrophage polarization (30), this study spatially resolves metabolic-immune crosstalk and highlights the MIF-CD44 axis as a plausible molecular bridge contributing to immune evasion. Spatial transcriptomics demonstrates the segregated yet contiguous localization of high-glycolytic C1 cells and macrophages, which interface directly at the tumor-stroma boundary. This configuration is consistent with a paracrine signaling axis in which C1 cells may secrete MIF to modulate peripheral macrophages. Although MIF is recognized for inhibiting macrophage migration and promoting survival (31, 32), our findings suggest that the malignant C1 clone may represent an important cytokine source. This suggests that intervening at the metabolic origin may prove as effective as conventional direct cytokine blockade.

Integration of scRNA-seq, bulk RNA-seq, and Mendelian randomization identified NEK6, a NIMA-related serine/threonine kinase critical for cell cycle control (33), as a causal risk factor for CRC. NEK6 dysregulation pervades numerous solid tumor types (e.g., breast, gastric, prostate, and ovarian cancers), where elevated expression is closely tied to tumor advancement, therapy resistance, and poor survival (19, 20, 34, 35). In hepatocellular carcinoma (HCC), this pronounced upregulation strongly predicts unfavorable clinical outcomes (36). Mechanistically, NEK6 drives HCC progression and metabolic reprogramming toward glycolysis by facilitating TCP10L ubiquitination and subsequent degradation (21). Consistent with these findings, our *in vitro* functional assays demonstrate that NEK6 downregulation significantly suppresses both proliferation and glycolytic activity. Furthermore, the co-localization of NEK6 and the C1 subtype in tumor regions supports its association with this malignant phenotype in spatial contexts. In addition to its association with glycolysis-related phenotypes, co-culture experiments suggest that NEK6 may also influence macrophage polarization. THP-1-derived macrophages co-cultured with CRC cells displayed increased CD206 expression, whereas this effect was attenuated following NEK6 knockdown in tumor cells. This observation is consistent with our single-cell and spatial analyzes, as well as immune cell infiltration analysis. Nevertheless, whether this effect is directly mediated by NEK6-dependent signaling or occurs secondarily to altered tumor cell metabolism or proliferation remains unresolved. Our multi-omics integration also identified UGT2B17 as a potential protective factor. As a phase II metabolic enzyme primarily involved in glucuronidation, its specific role in CRC remains largely uncharacterized.

While our findings suggest that NEK6-associated glycolytic phenotypes may represent a potential therapeutic vulnerability, the translational challenge must be critically evaluated. NEK6 maintains basal expression in normal proliferating tissues due to its essential role in mitotic progression and spindle formation. Consequently, systemic NEK6 inhibition could precipitate anti-mitotic toxicities, most notably bone marrow suppression and gastrointestinal mucosal damage. Any future attempt to therapeutically target NEK6 would require careful consideration of tumor specificity and systemic toxicity, particularly given its established role in normal proliferating tissues. Therefore, additional *in vivo* and pharmacological studies will be essential before any translational strategy can be meaningfully evaluated.

This study has several limitations. First, while our *in vitro* co-culture assays supported an association between NEK6 and M2 macrophage polarization, these static models cannot recapitulate the complex spatial and temporal dynamics of the *in vivo* tumor microenvironment. In addition, although a biological blank control was included in the flow cytometry analysis, a dedicated isotype-based gating control was not available, which may limit the rigor of the macrophage polarization readout. A major limitation of the present study is the absence of *in vivo* validation using animal models to definitively substantiate the effects of NEK6 depletion on CRC progression and immune remodeling. Furthermore, the clinical translation of these findings is currently limited by the lack of highly selective, clinical-grade pharmacological inhibitors targeting NEK6. Secondly, while our *in vitro* loss-of-function assays support the functional importance of NEK6 in maintaining glycolysis, the precise downstream mechanistic cascade remains to be fully elucidated. Additional mechanistic evidence will be necessary to establish its definitive hierarchical role in metabolic reprogramming. Importantly, the present study does not establish whether the observed reductions in ECAR, lactate production, ATP levels, and glycolysis-related proteins reflect a direct regulatory role of NEK6 in glycolysis or arise secondarily from its established functions in cell cycle progression and proliferation. Although three independent siRNAs were initially screened, the downstream functional assays were performed using a single siRNA, which may carry a risk of sequence-specific off-target effects. Further mechanistic studies, including validation with additional independent siRNAs and rescue experiments, will be required to disentangle these possibilities. Finally, our single-cell transcriptomic analysis was based on a relatively limited cohort of 13 samples. Although we robustly validated the clinical prognostic significance of NEK6 across multiple large, independent external cohorts, the single-cell findings derived from this restricted sample size may not fully capture the complete spectrum of metabolic and immune dynamics present in the broader CRC patient population.

In conclusion, this study characterizes the spatial and metabolic heterogeneity in CRC, demonstrating that the high-glycolytic Glycolysis-C1 subtype is associated with immune microenvironment remodeling via paracrine MIF signaling. Moreover, NEK6 is identified as a candidate gene associated with proliferative and glycolytic phenotypes in CRC. These findings suggest that NEK6 may represent a potential therapeutic vulnerability for future investigation. However, extensive *in vivo* validation and complementary mechanistic studies remain essential prerequisites before considering its clinical translation.

## Data Availability

Publicly available datasets were analyzed in this study. This data can be found here: GSE161277: https://www.ncbi.nlm.nih.gov/geo/query/acc.cgi?acc=GSE161277; GSE283052: https://www.ncbi.nlm.nih.gov/geo/query/acc.cgi?acc=GSE283052; GSE103479: https://www.ncbi.nlm.nih.gov/geo/query/acc.cgi?acc=GSE103479; GSE17536: https://www.ncbi.nlm.nih.gov/geo/query/acc.cgi?acc=GSE17536.
