# Supplementary figures and images for: Integrating single-cell and spatial transcriptomics reveals glycolysis heterogeneity and NEK6-mediated progression in colorectal cancer
Source: Front Immunol. 2026 Apr 28;17:1802329. doi: 10.3389/fimmu.2026.1802329 (PMC13161100; doi:10.3389/fimmu.2026.1802329)

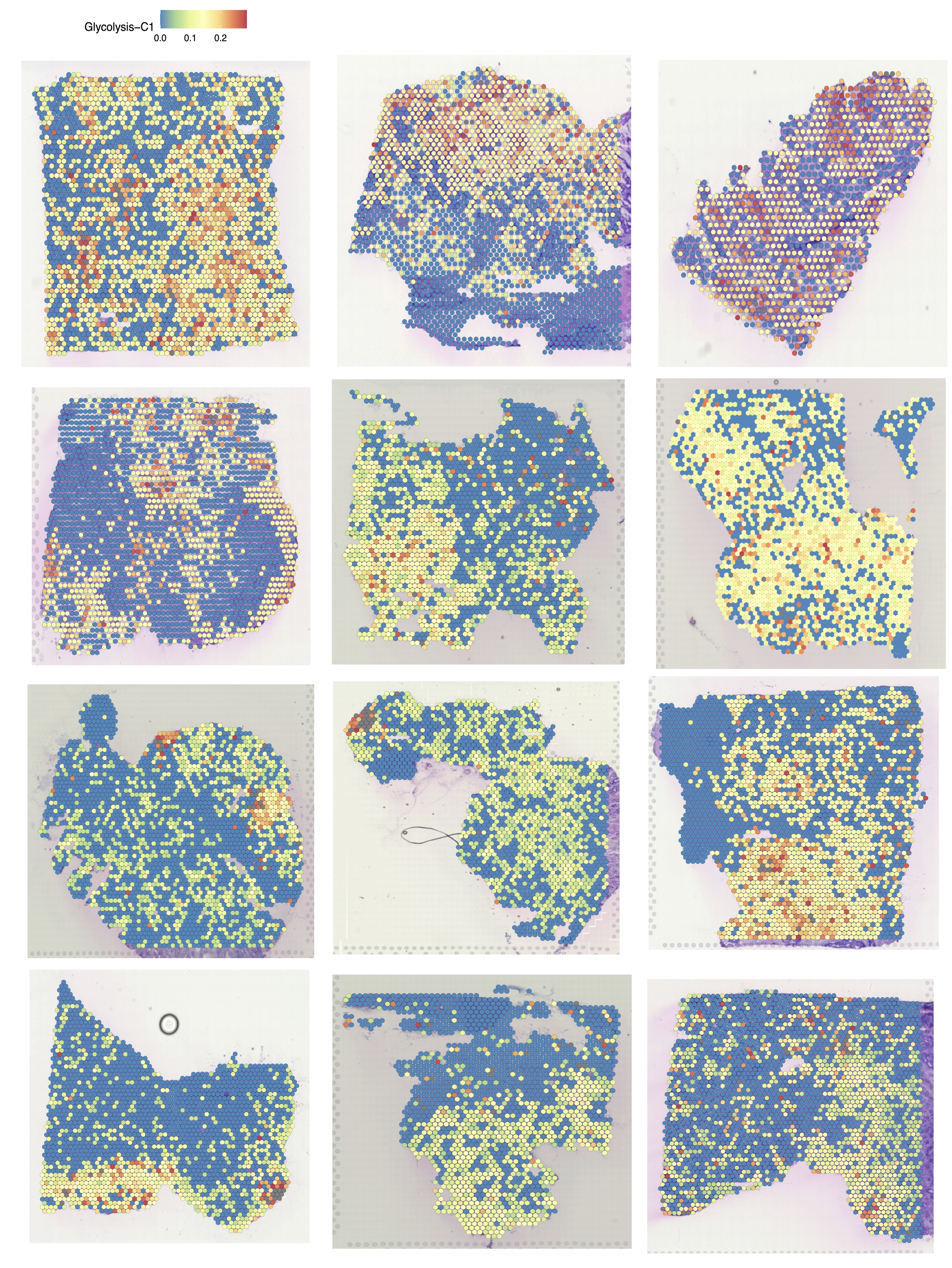

Supplement: Supplementary file 1 [file Image1.jpeg]

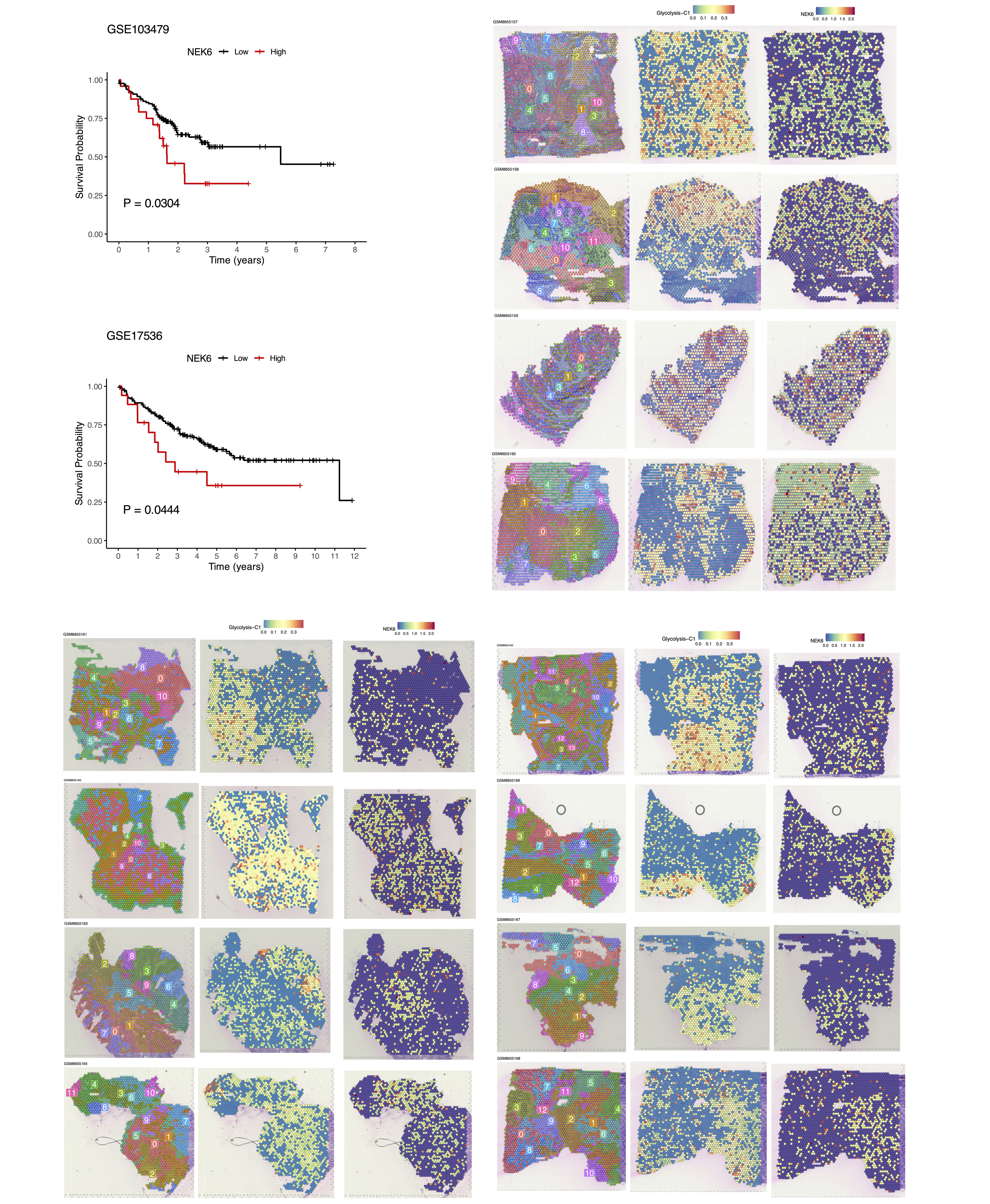

Supplement: Supplementary file 2 [file Image2.jpeg]

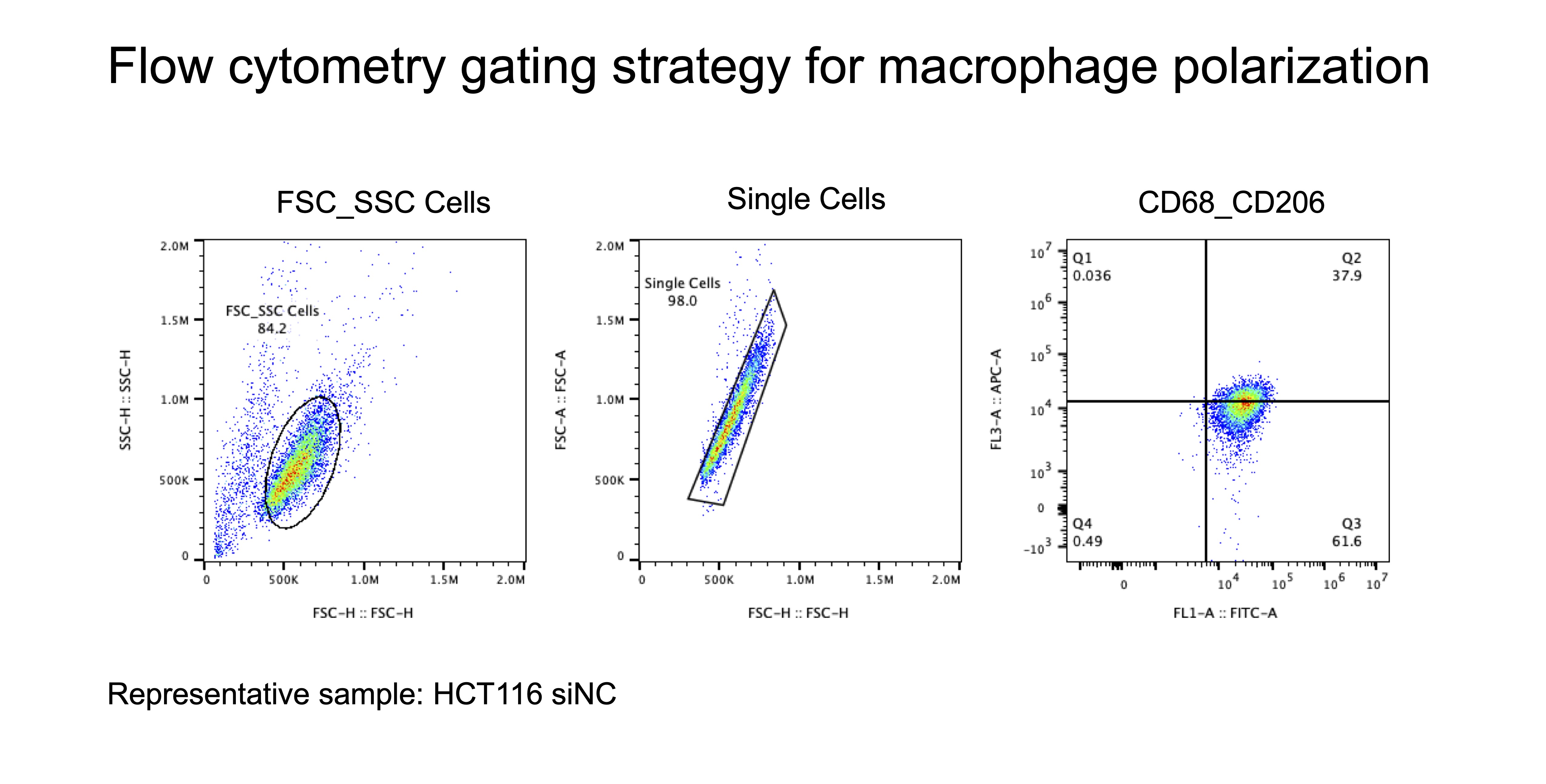

Supplement: Supplementary file 3 [file Image3.jpeg]
